# Publication Dynamics Where Evidence Is Missing: Mapping Empty Reviews in Nursing

**DOI:** 10.1111/wvn.70125

**Published:** 2026-02-28

**Authors:** Chiara Moreal, Gaia Magro, Daniela D'Angela, Renzo Moreale, Gaia Dussi, Sara Dentice, Stefania Chiappinotto, Alvisa Palese

**Affiliations:** ^1^ Department of Medicine University of Udine Udine Italy

**Keywords:** empty review, gaps, methodology, nursing, nursing research, publication bias, scoping review

## Abstract

**Introduction:**

The production of science is characterized by socio‐political and technological forces that influence what knowledge is produced. In this context, empty reviews have received little attention, with debate ranging over the pros and cons of their publication. However, their dissemination may improve the ability to recognize and prioritize research gaps. The main aim of the study was to map empty reviews published in nursing science.

**Materials and Methods:**

A scoping review in accordance with Arksey and O'Malley, Joanna Briggs Institute and Preferred Reporting Items for Systematic Reviews and Meta‐Analyses for Scoping Reviews. The review protocol was registered in the Open Science Framework database in April 2025. Four databases and grey literature were searched; there were eligible scoping or systematic reviews defined as “empty” in the field of nursing. A modified framework of Patterns, Advances, Gaps, Evidence for practice, and Research recommendations was used to summarize the extracted data.

**Results:**

Fifteen empty reviews were identified. In terms of Patterns, the empty reviews were mainly published in high‐income countries over the last 10 years and related to clinical practise and outcomes, education and training, organizational and human resources, and approaches to maternity care, mental health, and nursing education. In general, reporting guidelines were used, while funding was not documented. In terms of Recommendations, more primary studies, the development of tools and the strategic use of empty reviews to inform the funding and research agenda were suggested.

**Linking Evidence to Action:**

Empty reviews in nursing may indicate neglected or emerging areas that can help orient research agendas to ensure equity‐oriented priorities and reduce the marginalization of under‐investigated topics. Recognizing empty reviews as legitimate scholarly outputs supports transparent mapping of knowledge gaps, helping funders, institutions, and research programs direct resources to under‐investigated areas. Dedicated registries that publicly report empty reviews, establish minimum reporting standards, and require explicit keywords in titles and abstracts would improve transparency and accessibility, and stimulate targeted primary research that can turn “empty” areas into active inquiry. From this perspective, empty reviews may attract research investment rather than be seen as methodological failures.

## 1 Introduction

Scientific research does not take place in isolation, but is shaped by social, technological, and political factors, reflecting its inherently socio‐technical and politically motivated character (Florides and Christodoulides 2023). The processes of knowledge production, validation and dissemination are influenced by the internal needs and priorities of developing scientific disciplines and also by external forces such as technological innovation, regulatory frameworks and funding dynamics (Raynaud et al. 2021). In healthcare research, these forces increasingly influence the nature of evidence production (Peters et al. 2021) and also shape the knowledge that is recognized as valuable (Železnik et al. 2017).

In this landscape, empty reviews, where authors have not found suitable studies (Lang et al. 2007), are stimulating an interesting debate. Overall, an empty review does not only mean the absence of studies in a particular area. It can also reflect a new area of research or be a consequence of a very specific review question or strict methodological inclusion criteria (Yaffe et al. 2012). However, while Cooper asserted that research syntheses should address topics for which there is already a body of evidence (Cooper 2017), others have considered the meaningful indicators of evidence gaps—such as the empty review report—as a starting point for prioritizing research (Jones 2022).

Methodological authorities have emphasized the importance of identifying and transparently reporting empty reviews to ensure accurate evidence mapping and inform research agendas (Higgings et al. 2024; Peters et al. 2021). The Cochrane Empty Reviews Project has shown that empty reviews are relatively common in healthcare research. Overall, 8.7% of Cochrane systematic reviews were categorized as empty based on searches up to 2010, with an increasing trend in their publication over time (Yaffe et al. 2012). However, empty reviews appear to be rare in some specific discipline, such as nursing: An examination of 293 systematic reviews published in nursing journals found no empty reviews (Gray 2020), which contrasts with general patterns in healthcare, where evidence gaps are increasingly documented (Wang et al. 2022). The few empty reviews available in nursing may reflect editors' reluctance to publish given their perceived low impact leading to systematic publication bias (Gray 2021; Slyer 2016). This may also be due to the prioritization of primary and secondary studies that document positive outcomes as also influenced by impact metrics and citation incentives that systematically marginalize the reporting of negative or null results (Pollock et al. 2023). In addition, funding can also influence publication trends: Between 2008 and 2018, funded publications in nursing research more than doubled, with a concentration in high‐priority areas such as oncology and deemphasizing other areas (Zhu et al. 2021). Consequently, wider shifts in publication dynamics may also influence the publications of empty reviews, where citation‐related incentives and funding adjustments determine what should be published and recognized as valuable knowledge (Gray 2021). Furthermore, recent bibliometric analyses have shown that articles recognized as exemplary in nursing tend to reinforce established research pathways, further entrenching selective visibility and narrowing the perceived landscape of nursing scholarship (Nicoll et al. 2020). Assessing empty reviews as legitimate scientific outputs could instead help to accurately map knowledge gaps and guide responsible research investment (Higgings et al. 2024; Jones 2022) by providing a transparent account of neglected areas and helping to counter publication bias that distorts perceptions of research maturity and progress (Wells et al. 2018). However, despite their relevance, to our knowledge no summary of empty reviews published in the field of nursing has been produced to date. In the broader context of publication dynamics, our intent is to summarize empty reviews published in nursing to provide insights into their patterns, the current state of knowledge gaps, and the research recommendations.

### 1.1 Objective of the Study

The aim was to identify, map, and analyze empty reviews published in the nursing field.

## 2 Materials and Methods

A scoping review was conducted in 2025, following the methodological framework of Arksey and O'Malley (2005) and the Joanna Briggs Institute Reviewers' Manual (JBI) (Aromataris et al. 2024). The study design was chosen with the intention of reflecting studies that were conducted in a comprehensive manner (Peters et al. 2021) with a systematic, transparent and replicable process. The following steps were ensured: (a) research question formulation and (b) relevant studies identification; (c) study selection, (d) data summarization and (e) synthesis of results. The review adhered to the Preferred Reporting Items for Systematic Reviews and Meta‐Analyses and its extension for scoping reviews (Tricco et al. 2018) (Table S1). In addition, the research protocol was registered in the Open Science Framework database (https://osf.io/kxzb4/overview) on 8 April 2025.

### 2.1 Stage 1. Research Question

The research question was: “Which empty reviews have been published in the field of nursing?” The Population, Concept and Context (PCC) methodological approach was used to define the components of the review question and to approach the literature search (Pollock et al. 2023). The population (P) referred to “empty review,” the concept (C) to the “absence of studies,” and the context (C) to the “nursing field.”

### 2.2 Stage 2. Relevant Studies

To identify the relevant studies, the 3‐step search process of the JBI (Aromataris et al. 2024) was applied.

In the first step, an initial exploratory search was conducted in PubMed and the Cumulative Index to Nursing and Allied Health Literature (CINAHL) to identify “seed references” that are defined as records of studies that meet the inclusion criteria for the review question (Aromataris et al. 2024). To improve the search strategy, additional searches were conducted using Google and Google Scholar. Furthermore, in consultation with the researchers (CM, DD, GM, SC, AP), the list of keywords and indexed terms was refined to ensure a comprehensive and well‐structured search framework.

In the second step, the relevant Medical Subject Headings (MeSH) terms and keywords were combined using truncation symbols and Boolean operators: “nurs*,” “review,” “empty review,” “no result,” “no eligible,” and “nursing” (Table S2). The search was conducted in four databases: PubMed, Scopus, CINHAL and Cochrane Database of Systematic Review. To mitigate potential publication and information bias, particularly given the underrepresentation of empty reviews (Slyer 2016), the grey literature was systematically searched by manually reviewing a selected list of relevant websites.

In the third step, the reference lists of the included studies were analyzed to identify further eligible studies. The guidelines of the Terminology, Application, and Reporting of Citation Searching (TARCiS) statement (Hirt et al. 2024) were followed, which emphasize backward and forward citation searching as essential complementary search techniques.

No restrictions were placed on language or publication date to avoid language bias and ensure comprehensive inclusion of studies (Aromataris et al. 2024). Details of the search strategy, including databases and sources, can be found in Table S3.

### 2.3 Stage 3. Study Selection

There were eligible secondary studies in the field of nursing that did not retrieve any studies to include, thus reflecting an empty review as defined by Lang et al. (2007). Included were (1) scoping or systematic reviews that were explicitly defined as “empty” and (2) that focused on nursing. Therefore, the following were excluded: (1) reviews that included at least one study; (2) methodological or theoretical papers, abstracts, and letters to the editor; and (3) studies not related to the field of nursing.

RAYYAN software (Ouzzani et al. 2016) was used to organize the retrieved sources and make the screening process efficient. First, duplicates were removed. Then, the titles and abstracts of the studies were screened by three independent reviewers (CM, DD, GM) based on the eligibility criteria; in case of disagreement, two additional reviewers (AP, SC) were consulted until a consensus was reached. The full texts of the studies were then retrieved to determine eligibility. At the end of each phase of the screening process, meetings were held between the reviewers, and disagreements were discussed until a consensus was reached or the issue was resolved.

### 2.4 Stage 4. Data Extraction

Based on the research question, key data from the included studies were captured in a table developed, reviewed, and refined by two reviewers (CM, GM) under the supervision of scientific experts (SC, AP). The data extraction form included: (1) general information (title, authors, year, publisher or repository, and country of affiliation of the authors) and funding related to the financial support (or lack thereof) reported by the authors (Álvarez‐Bornstein and Montesi 2021); (2) study design (aim, type of study, methodological guidelines followed, protocol registration, and consultation with a librarian); (3) search process (databases and registries accessed, other search sources, number of studies initially included, reviewed in full text, and finally included); (4) main findings (documented reasons for absence of studies, limitations, description of excluded studies, and recommendations for future research). Data extraction was performed independently by three reviewers (CM, DD, GM) and then combined. In case of discrepancies, two additional researchers were consulted (AP, SC).

### 2.5 Stage 5. Data Collection, Summary and Report of the Results

A modified PAGER framework (Bradbury‐Jones et al. 2022) was used to organize the results according to Patterns, Advances, Gaps, Evidence for practice, and Research recommendations, allowing for a structured and comprehensive synthesis based on the research question. Specifically, in line with the nature of the study, which was to map the available empty reviews, the Advances and Evidence were not summarized. The findings extracted from the retrieved empty reviews were separated and then categorized following an inductive analysis (Ahmed et al. 2025). The Patterns, Gaps, and Recommendations were then mapped. Three researchers (CM, DD, GM) conducted the process, which was subsequently checked by the rest of the research team (see authors).

## 3 Results

### 3.1 Flow of Results

**FIGURE 1:**
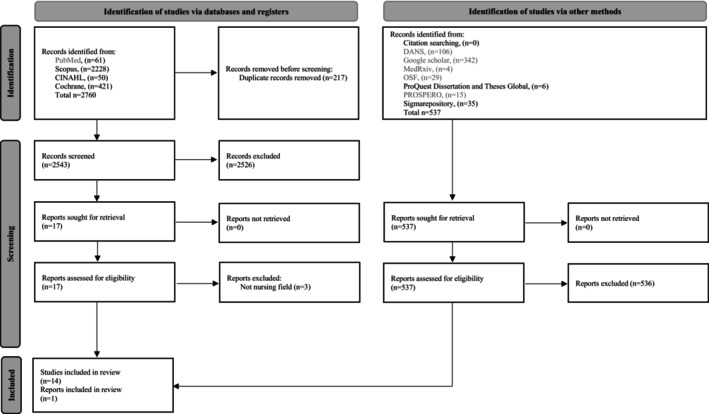
PRISMA 2020 flow diagram for new systematic reviews which included searches of databases, registers and other sources (Page et al. 2021). CINAHL, Cumulative Index to Nursing and Allied Health Literature; DANS, Data Station Life Science; OSF, Open Science Framework; PRISMA, Preferred Reporting Items for Systematic Reviews and Meta‐Analyses; PROSPERO, International prospective register of systematic reviews.

Based on the flowchart of the Preferred Reporting Items for Systematic reviews and Meta‐Analyses (PRISMA) 2020 (Page et al. 2021), the results of the search process are shown in Figure 1. Initially, 3297 records were identified from all sources. After excluding studies that did not fulfill the eligibility criteria, 18 full‐text articles were reviewed, of which three were excluded (Bercier and Maynard 2015; Rosati et al. 2024; Sheikh et al. 2008) (Table S4). The reference lists of the included studies were also checked; however, no further relevant reviews were identified. Thus, a total of 15 empty reviews were retrieved, including one from grey literature.

As summarized in Table 1, overall, studies were published between 2015 (Chang et al. 2015) and 2025 (Jonckers et al. 2025). The first authors were mainly affiliated in the United States (*n* = 5; Bertocchi et al. 2025; Brown et al. 2021; Johnson et al. 2024; Leighton et al. 2021; Sibley et al. 2024) and Australia (*n* = 5; Chang et al. 2015; Coyne et al. 2016; Hanna et al. 2023; Heffernan et al. 2022; Moyo et al. 2020). Four were published in the Cochrane Database of Systematic Reviews (Chang et al. 2015; Coyne et al. 2016; Hanna et al. 2023; Moore et al. 2016), while each of the other journals contributed only a single report. Five studies (e.g., Bertocchi et al. 2025) declared in the title that the review was “empty.”

**TABLE 1:** Data extraction of included empty reviews (*n* = 15).

Author (year)	Aim	Databases/Registries	Limitations
Authors' affiliation countries	Review type	Others search sources	Reasons for empty
Title	Methodological guideline	Initial studies found, *n*	Recommendations for future research
Journal/repository	Protocol's registration	Full text studies included, *n*	
Funding	Librarian consultation	Final studies included, *n*	
		Description of excluded studies	
Bertocchi et al. (2025) Italy, USA Exploring the nexus between the standardized nursing terminologies and the unfinished nursing care phenomenon: An empty systematic review *International Journal of Nursing Knowledge* No	To identify and synthesize evidence concerning the documented relationship between the SNTs and the UNC phenomenon Systematic review PRISMA guidelines Not registered NR	CINAHL, PubMed, Scopus Contacting experts, reference lists 149 1 0 Wrong evaluation (no clear association between SNTs and UNC)	Restricted eligibility criteria in language (English, Italian); database selection; complexity in integrating SNTs and UNC conceptual frameworks Lack of studies linking SNTs and UNC To conduct empirical studies linking SNTs and UNC; to explore the integration of digital systems and clinical terminologies to detect, document, and prevent UNC
Brown et al. (2021) USA Association between nurse work environment and severe maternal morbidity in high‐income countries: A systematic review and call to action *Journal of Advanced Nursing* NR	To synthesize and appraise research regarding the association between nurse work environment and SMM in HICs Systematic review PRISMA guidelines PROSPERO Yes	CINAHL, Cochrane Database of Systematic Reviews, PubMed, Scopus Citation tracking 739 159 0 Wrong outcome (association between nurse work environment and non‐maternal patient health, nurse‐reported non‐maternal patient, nurse job, nursing care processes, nurse‐reported quality of care)	Restricted eligibility criteria in setting (high‐income countries), language (English), time frame (1990–2019); human error (overlooking during screening) Lack of knowledge about SMM as a patient outcome in the nurse work environment To investigate the relation between the nurse work environment and SMM in HICs; to conduct high‐quality cross‐sectional and longitudinal studies and use theoretical frameworks; to highlight the value of empty reviews in identifying funding priorities
Chang et al. (2015) Australia, Malaysia, Singapore Specialist teams for neonatal transport to neonatal intensive care units for prevention of morbidity and mortality (Review) *Cochrane Database of Systematic Reviews* Yes, Newborn and pediatric Emergency Transport Service, Australia; Centre for Perinatal Health Services Research, Australia; SEA‐ORCHID, Australia; National Health and Medical Research Council, Australia; Wellcome Trust, UK; Eunice Kennedy Shriver National Institute of Child Health and Human Development National Institutes of Health, Department of Health and Human Services, USA	To examine the evidence as to whether specialist teams for neonatal transport to neonatal intensive care units results in better outcomes and decreases mortality and morbidity among newborn infants Systematic review Criteria and standard methods of Cochrane and the CNRG Cochrane registry No	Australian New Zealand Clinical Trials Registry, Clinicaltrials.gov, CINAHL, Cochrane Library, EMBASE, ISRCTN registry, PubMed, WHO's ICTRP Reference list, experts in the field NR 1 0 Risk of bias (subsequent withdrawal of a identified study from publication)	NR Lack of availability of the specialist team transport, ethical, logistical, and technical challenges To conduct cluster RT among different specialist team members regarding clinical outcomes and effectiveness of specialist‐trained neonatal transport teams
Coyne et al. (2016) Australia, Ireland, Netherlands, UK Interventions for promoting participation in shared decision making for children with cancer (Review) *Cochrane Database of Systematic Reviews* Yes, Stichting Kinderen Kankervrij, Netherlands	To examine the effects of SDM interventions on the process of SDM for children with cancer who are aged four to 18 years Systematic review Cochrane Handbook for Systematic Reviews of Interventions Cochrane registry NR	BIOSIS, CINAHL, Cochrane Library, EMBASE, ERIC, ProQuest Dissertations and Theses, ProQuest Sociological Abstracts, PsycINFO, PubMed AACH, Annual Conference of SIOP, Annual Scientific Meeting of SMDM, EACH, ECCO, ESMO, ICCH, ISDM, ISRCTN, NIH 3290 1 0 Wrong population (not during cancer treatment), wrong outcome (treatment adherence knowledge, cancer‐specific self‐efficacy, quality of life, stress and control)	NR Lack of studies evaluating the effects of interventions that promote participation in SDM for children with cancer To conduct RCT to investigate the effects of interventions that promote participation in SDM for children with cancer; to identify how new multi‐media innovations can support information exchange between children and healthcare professionals
Hanna et al. (2023) Australia, USA Infant isolation and cohorting for preventing or reducing transmission of healthcare‐associated infections in neonatal units (Review) *Cochrane Database of Systematic Reviews* Yes, Vermont Oxford Network	To assess the effectiveness of single‐room isolation or cohorting, or both, for preventing HAIs or colonization in neonates in NICUs Systematic review Cochrane Neonatal Cochrane registry Yes	CINAHL, Cochrane Library, EMBASE, PubMed, US National Library of Medicine's, WHO's ICTRP PAS conference website 2044 7 0 Wrong design (not randomized), wrong outcome (not allow the utility of isolation and cohorting)	NR Lack of interventions that include isolation or cohorting To conduct well‐designed randomized studies, both cluster‐randomized and individually randomized; to evaluate the effectiveness, safety, and cost‐effectiveness of patient isolation measures
Harrison et al. (2023) UK Unobtrusive and acceptable ways to gather participant perceptions of community‐based interventions and their effectiveness at improving mental health and wellbeing: A literature review of peer reviewed and grey literature *Health & Social Care in the Community* Yes, National Institute for Health and Care Research	To identify unobtrusive methods to examine service user's perceptions of community‐based mental health interventions and their effectiveness, and the perceived acceptability of the methods Systematic review PRISMA guidelines Research Registry (review registry 1401) No	EMBASE, ProQuest Dissertations and Theses Global, PsycINFO, PubMed, Web of Science Google, known experts 930 12 0 Wrong methods (surveys, interviews, focus groups), wrong intervention (on perceptions)	Restricted eligibility criteria in setting (community‐based), language (English) Lack of innovative and unobtrusive data collection methods to assess perceptions, unsuitability of unobtrusive methods to the context of community‐based interventions To conduct studies considering the development or adaptation of existing methods to gather perceptions of the effectiveness of service users; to generate evidence to support funding of community‐based interventions
Heffernan et al. (2022) Australia, UK Tri‐response police, ambulance, mental health crisis models in reducing involuntary detentions of mentally ill people: A systematic review *Nursing Reports* No	To synthesize the available evidence regarding the effects of the tri‐response police, ambulance, mental health crisis model in diverting patients from hospital and reducing unnecessary involuntary detention Systematic Review PRISMA guidelines OSF Yes	CINAHL, ProQuest, PsycINFO, PsychArticles, PubMed Google, Open Gray 239 1 0 Wrong intervention (tri‐response mental health crisis models: police + ambulance + MH clinician)	Restricted eligibility criteria in design of review (systematic vs. scoping review), language (English); inability to access policing databases, inclusion of grey literature Lack of evidence regarding the efficacy of tri‐response mental health crisis models (police + ambulance + MH clinician) To conduct robust studies specifically on tri‐response models to assess their impact on reducing involuntary detentions and improving emergency mental health care
Johnson et al. (2024) USA Advanced practice nurses' preparation: transitioning from clinical practice to academia: A scoping review *Sigma Repository* No	To identify what was known about the preparation of APRNs when transitioning from clinical practice to the online/hybrid academic setting Scoping review PRISMA‐ScR NR Yes	MEDLINE, CINAHL, ProQuest Nursing and Allied Health Literature, ProQuest Education Reference list 4387 11 0 Wrong publication type (not peer‐reviewed), wrong population (not APRNs), wrong outcome (not on transitioning from clinical practice to academia)	Restricted eligibility criteria in time frame (2014–2024), language (English); exclusion of grey literature Lack of studies specifically addressing the transition of APRNs from clinical practice to online/hybrid academia To conduct high‐quality research studies exploring the transition of APRNs from clinical practice to online/hybrid academia and strategies to support it
Jonckers et al. (2025) Netherlands Humidification of incubator air for premature infants: An empty systematic review *Journal of Neonatal Nursing* No	To review the effects of low vs. moderate/high humidity and short vs. long durations of humidity exposure on preterm infants in incubators Systematic Review PRISMA guidelines NR Yes	Cochrane Library, EMBASE, Ovid Emcare, PubMed, Web of Science No 1124 4 0 Wrong outcome (e.g., studied sleep, behavior, metabolic costs) wrong comparison (not comparing humidity levels or durations as defined)	Restricted eligibility criteria in language (English and Dutch) Lack of evidence in the humidification air of incubator To conduct studies comparing humidity levels and durations in incubators; to define outcomes like thermoregulation, skin integrity, infections
Leighton et al. (2021) Canada, Qatar, USA Traditional Clinical outcomes in prelicensure nursing education: An empty systematic review *Journal of Nursing Education* NR	To examine the evidence regarding the effectiveness of traditional clinical experiences in achieving student learning outcomes in prelicensure nursing education Systematic Review JBI and PRISMA guidelines NR Yes	CENTRAL, CINAHL Plus, Cochrane Database of Systematic Reviews, ERIC, Global Health, JBI Evidence‐Based Practice Database, PsycINFO, PubMed, Scopus No 1239 118 0 Wrong methodology (poor, unvalidated tools), wrong concept (mismatch, self‐perceptions), wrong outcome (not actual)	Restricted eligibility criteria in language (english) Lack in the evaluation of students in the clinical setting To conduct scientific research and test alternate models in nursing education for increasingly complex healthcare environments and patient care; to develop further studies in interprofessional learning and practice; to find available funding to support rigorous inquiry
Moore et al. (2016) Ireland, Norway, UK Bed rest for pressure ulcer healing in wheelchair users (Review) *Cochrane Database of Systematic Reviews* Yes, Royal College of Surgeons in Ireland, Ireland; School of Nursing, Midwifery and Social Work, University of Manchester, UK; National Institute for Health Research, via Cochrane Infrastructure and Cochrane Programme Grant funding to Cochrane Wounds	To assess the impact of bed rest on pressure ulcer healing in wheelchair users of any age, in any care setting Systematic Review Cochrane Handbook guidelines Cochrane registry Cochrane Highly Sensitive Search Strategy	CENTRAL, Clinicaltrials.gov, CINAHL Plus, Cochrane Wounds Register, EMBASE, EU Clinical Trials Register, MEDLINE, WHO ICTRP Conference proceeding, reference list 73 0 0 Wrong design (no RCTs or cluster‐RCTs)	NR Lack of trials regarding the impact of bed rest on pressure ulcer healing in wheelchair users To conduct well‐designed RCTs; to ensure sufficiently large sample sizes; to include patient‐centered outcomes (treatment acceptability, adverse events, health‐related quality of life); to incorporate economic evaluations; to use standardized and validated outcome measures
Moyo et al. (2020) Australia The association between the mental health nurse‐to‐registered nurse ratio and patient outcomes in psychiatric inpatient wards: A systematic review *International Journal of Environmental Research and Public Health* Yes, doctoral studentship from La Trobe University	To synthesize evidence examining the association between the mental health nurse‐to‐registered nurse ratio and psychiatric readmission or crisis referral in adult inpatients Systematic Review PRISMA guidelines PROSPERO NR	CINAHL, Cochrane Central, EMBASE, MEDLINE, PsycINFO No 7956 4 0 Wrong population (industrial action, nursing skill mix, psychiatrics and nurse staffing), wrong outcome (conflict, containment, seclusion)	Restricted eligibility criteria in design of review (systematic vs. scoping review), language (English); exclusion of grey literature; measurable valid outcome in mental health research Lack in high‐quality skill‐mix research to determine optimal and safe staffing in mental‐inpatient settings; planning different study design (systematic vs. scoping review) To conduct rigorous observational and experimental studies on skill mix in mental health inpatient settings; to explore appropriate outcomes such as readmission or untoward incidents
Sibley et al. (2024) USA Effectiveness of objective structured clinical examinations and standardized patient simulations for increasing learner knowledge in family nurse practitioner education: A systematic review of the literature *Nursing Education Perspectives* NR	To evaluate the effectiveness of OSCEs and SP simulations in FNP education for increasing student knowledge and skill competency Systematic Review PRISMA guidelines PROSPERO Yes	CINAHL, Cochrane, ERIC, ProQuest Dissertations and Theses Global, PsycINFO, PubMed, Web of Science AACN, ASPE, CADTH GrayMatters, INACSL, MedNar, medRxiv, NLN, OCLC, online conference sites in nursing education, SSH, WorldCat dissertations 5245 52 0 Wrong population (not FNP), wrong design (lack of comparison group), wrong outcome (not measuring outcomes of interest)	Restricted eligibility criteria in design (comparative studies only), population (Family Nurse Practitioner students), intervention (OSCE and SP simulation only), outcome (learner knowledge and skill performance), language (English) Lack of comparative quantitative studies focused on FNP students that measured knowledge or skill outcomes To conduct comparative, rigorous RCTs on OSCE and SP simulations in FNP education; to ensure large sample sizes; to build evidence to influence policy and practice in competency‐based education; to support funding opportunities addressing critical gap in nursing
Tzenetidis et al. (2023) Greece The relationship between psychosocial work environment and nurses' performance, on studies that used the validated instrument Copenhagen psychosocial questionnaire (COPSOQ): an empty scoping review *Polish Medical Journal* NR	To explore the relationship between psychosocial work environment and nurses' performance in studies using the COPSOQ instrument Scoping review PRISMA‐ScR, JBI NR NR	MEDLINE, Science Direct Google Scholar, COPSOQ International Network 15,108 68 0 Wrong results (insufficient), wrong intervention (partly use of COPSOQ)	Restricted eligibility criteria in time frame (2000–2021), language (English); omission of surveys using partial questionnaires Lack of research regarding the psychosocial component of nurses in its entire dimension To use the entire COPSOQ questionnaire to fully assess psychosocial risks among nurses; to include studies in additional languages; to consider partial‐use studies
Walsh et al. (2021) Canada Nurses' decision‐making related to administering as needed psychotropic medication to persons with dementia: an empty systematic review *International Journal of Older People Nursing* NR	To systematically review the literature regarding nurses' decision‐making in administering PRN psychotropic medication to persons with dementia in acute care settings Systematic review PRISMA flowchart NR Yes	CINAHL, EMBASE, MEDLINE, Scopus, ProQuest Dissertations and Theses Global Reference list 677 43 0 Wrong setting (not hospitalized), wrong aim (inappropriate), wrong population (children, people with dementia), wrong intervention (not related to medication administration or decision‐making)	Restricted eligibility criteria in language (English) Lack of literature exploring nurses' decision‐making regarding PRN psychotropic medication for hospitalized persons with dementia in acute care settings To conduct studies on nurses' decision‐making for psychotropic use in acute care; to evaluate effects of educational interventions on PRN administration; to investigate links between patient safety and PRN use; to explore alternative non‐pharmacological strategies

Abbreviations: AACH, American Academy on Communication in Healthcare; AACN, American Association of Critical‐Care Nurses; APRN, Advanced Practice Registered Nurse; ASPE, Association of Standardized Patient Educators; BIOSIS, BioSciences Information Service of Biological Abstracts; CADTH, Canadian Agency for Drugs and Technologies in Health; CENTRAL, Cochrane Central Register of Controlled Trials; CINAHL, Cumulative Index to Nursing and Allied Health Literature; CNRG, Cochrane Neonatal Review Group; COPSOQ, Copenhagen Psychosocial Questionnaire; EACH, European Association for Communication in Healthcare; ECCO, European Cancer Organization; EMBASE, Excerpta Medica dataBASE; ERIC, Education Resources Information Center; ESMO, European Society for Medical Oncology; EU, European Union; FNP, Family Nurse Practitioner; HAI, Healthcare‐Associated Infections; HICs, High‐Income Countries; ICCH, International Conference on Communication in Healthcare; ICTRP, International Clinical Trials Registry Platform; INACSL, International Nursing Association of Clinical Simulation and Learning; ISDM, International Shared Decision Making Conference; ISRCTN, International Standard Randomized Controlled Trial Number; JBI, Joanna Briggs Institute; MEDLINE, Medical Literature Analysis and Retrieval System Online; *n*, number; NETS, Newborn & Pediatric Emergency Transport Service; NICU, Neonatal Intensive Care Unit; NIH, National Institutes of Health; NLN, National League for Nursing; NR, not reported; OCLC, Online Computer Library Center; OSCE, Objective Structured Clinical Examinations; OSF, Open Science Framework; PAS, Pediatric Academic Societies; PRISMA, Preferred Reporting Items for Systematic Reviews and Meta‐Analyses; PRISMA‐ScR, Preferred Reporting Items for Systematic Reviews and Meta‐Analyses Extension for Scoping Reviews; PRN, pro re nata; RCT, randomized controlled trial; RT, randomized trial; SDM, shared decision‐making; SIOP, International Society for Pediatric Oncology; SMDM, society for medical decision making; SMM, severe maternal morbidity; SNTs, standardized nursing terminologies; SP, standardized patient; SSH, Society for Simulation in Healthcare; UK, United Kingdom; UNC, unfinished nursing care; USA, United States of America; WHO, World Health Organization.

All reviews included a brief description of the reasons for the excluded studies that led to an empty review. A mismatch between the population, intervention, comparison, or outcome of the screened studies and the aims of the reviews or the eligibility criteria is the most common reason for exclusion. Moreover, the main reasons cited were the lack of randomized controlled trials, robust observational studies, or the prevalence of qualitative designs that did not meet the inclusion criteria (Chang et al. 2015; Coyne et al. 2016; Hanna et al. 2023; Moore et al. 2016). Two reviews excluded studies due to an unclear or insufficient conceptual link between the investigated constructs (Bertocchi et al. 2025; Leighton et al. 2021). Finally, one review reported the exclusion of a study due to a high risk of bias identified after publication, which led to a formal retraction of the study (Chang et al. 2015).

Among the limitations, language was most frequently cited, with most reviews restricting their search to English and, in some cases, Italian or Dutch. In contrast, only four reviews, all conducted by Cochrane groups, did not impose language restrictions (Chang et al. 2015; Coyne et al. 2016; Hanna et al. 2023; Moore et al. 2016). Time restrictions were only identified as limitations in three reviews (Brown et al. 2021; Johnson et al. 2024; Tzenetidis et al. 2023). Moreover, the exclusion of grey literature was mentioned as a limitation in two studies (Johnson et al. 2024; Moyo et al. 2020), while one study explicitly mentioned the inclusion of grey literature as a limitation due to data collection challenges (Heffernan et al. 2022). Selection bias was mentioned as a potential limitation due to human error during the screening process (Brown et al. 2021) and the databases selected (Bertocchi et al. 2025). Another limitation was the inaccessibility of certain policy documents, as reported by Heffernan et al. (2022). Two systematic reviews also acknowledged limitations in their study design, pointing out that conducting a scoping review instead of a systematic review could have provided more comprehensive results, potentially avoiding empty reviews (Heffernan et al. 2022; Moyo et al. 2020).

### 3.2 Patterns

Five main patterns emerged in relation to (1) methodological aspects (study design, reporting guidelines, pre‐registration and databases), (2) topics considered, and (3) funding (Table 2).

**TABLE 2:** Patterning chart of included empty reviews according to the modified PAGER framework (Bradbury‐Jones et al. 2022).

	Methodology							Topics				Funding
	Type of review		PRISMA guidelines	Cochrane guidelines	JBI guidelines	Review protocol registered	Number of databases and registries accessed	Clinical practice and outcomes	Concepts	Education and training	Organizational and human resources	
	Systematic	Scoping										
Bertocchi et al. (2025)	√		√				3		√			
Brown et al. (2021)	√		√			√	4	√				
Chang et al. (2015)	√			√		√	8	√				√
Coyne et al. (2016)	√			√		√	9	√				√
Hanna et al. (2023)	√			√		√	6	√				√
Harrison et al. (2023)	√		√			√	5	√				√
Hefferan et al. (2022)	√		√			√	5				√	
Johnson et al. (2024)		√	√				4			√		
Jonckers et al. (2025)	√		√				5	√				
Leighton et al. (2021)	√		√		√		9			√		
Moore et al. (2016)	√			√		√	8	√				√
Moyo et al. (2020)	√		√			√	5	√				√
Sibley et al. (2024)	√		√			√	7			√		
Tzenetidis et al. (2023)		√	√		√		2				√	
Walsh et al. (2021)	√		√				5	√				

Abbreviations: JBI, Johanna Briggs Institute; PAGER, Patterns, Advances, Gaps, Evidence for practice, and Research recommendations; PRISMA, Preferred Reporting Items for Systematic Reviews and Meta‐Analyses.

Firstly, 13 of the 15 studies were systematic reviews and two were scoping reviews. In addition, the PRISMA guidelines (Page et al. 2021) or related reporting tools were followed by the majority of the empty reviews (*n* = 11; Bertocchi et al. 2025; Brown et al. 2021; Harrison et al. 2023; Heffernan et al. 2022; Johnson et al. 2024; Jonckers et al. 2025; Leighton et al. 2021; Moyo et al. 2020; Sibley et al. 2024; Tzenetidis et al. 2023; Walsh et al. 2021). Four (Chang et al. 2015; Coyne et al. 2016; Hanna et al. 2023; Moore et al. 2016) were developed in accordance with the Cochrane standards, while two further reviews (Leighton et al. 2021; Tzenetidis et al. 2023) additionally referenced the methods recommended by the JBI.

Secondly, almost half of the reviews formally registered their protocols. Cochrane was the most common registry (*n* = 4; Chang et al. 2015; Coyne et al. 2016; Hanna et al. 2023; Moore et al. 2016), followed by PROSPERO (*n* = 3; Brown et al. 2021; Moyo et al. 2020; Sibley et al. 2024), the Research Registry (*n* = 1; Harrison et al. 2023), and OSF (*n* = 1; Heffernan et al. 2022).

Third, the number of databases and registries consulted ranged from a minimum of two (Tzenetidis et al. 2023) to a maximum of nine (Coyne et al. 2016; Leighton et al. 2021). CINHAL was the most frequently searched database (*n* = 12; Bertocchi et al. 2025; Brown et al. 2021; Chang et al. 2015; Coyne et al. 2016; Hanna et al. 2023; Heffernan et al. 2022; Johnson et al. 2024; Leighton et al. 2021; Moore et al. 2016; Moyo et al. 2020; Sibley et al. 2024; Walsh et al. 2021), followed by PubMed (*n* = 10; Bertocchi et al. 2025; Brown et al. 2021; Chang et al. 2015; Coyne et al. 2016; Hanna et al. 2023; Harrison et al. 2023; Heffernan et al. 2022; Jonckers et al. 2025; Leighton et al. 2021; Sibley et al. 2024). The registries were also frequently consulted, such as the WHO ICTRP (Chang et al. 2015; Hanna et al. 2023; Moore et al. 2016), the Cochrane Central Register of Controlled Trials (Leighton et al. 2021; Moore et al. 2016) and the Clinicaltrial.gov Register (Chang et al. 2015; Moore et al. 2016). Grey literature was searched in specific databases such as ProQuest Dissertation and Theses (Coyne et al. 2016; Sibley et al. 2024; Walsh et al. 2021), Google (Harrison et al. 2023; Heffernan et al. 2022; Tzenetidis et al. 2023) or nursing associations (Coyne et al. 2016; Sibley et al. 2024). One third of the included reviews reported an additional search strategy by consulting the reference list (*n* = 5; Bertocchi et al. 2025; Chang et al. 2015; Johnson et al. 2024; Moore et al. 2016; Walsh et al. 2021). In addition, collaboration with professional librarians or following established search instructions was explicitly described in the majority of reviews (*n* = 10; Brown et al. 2021; Chang et al. 2015; Hanna et al. 2023; Heffernan et al. 2022; Johnson et al. 2024; Jonckers et al. 2025; Leighton et al. 2021; Moore et al. 2016; Sibley et al. 2024; Walsh et al. 2021). One report explicitly declared that no librarians were involved (Harrison et al. 2023), while four did not specify this aspect (Bertocchi et al. 2025; Coyne et al. 2016; Moyo et al. 2020; Tzenetidis et al. 2023).

Fourth, the retrieved empty reviews related mainly to “Clinical Practise & Patient Outcomes” in terms of the topics considered (*n* = 9; Brown et al. 2021; Chang et al. 2015; Coyne et al. 2016; Hanna et al. 2023; Jonckers et al. 2025; Moore et al. 2016; Moyo et al. 2020; Walsh et al. 2021) exploring the effectiveness of clinical interventions or the association between nursing practises and patient outcomes. The main target groups were young children and people with mental health problems. Then “Education & Training” was also considered (*n* = 3; Johnson et al. 2024; Leighton et al. 2021; Sibley et al. 2024), focusing on educational strategies, assessment tools and training methods aimed at improving the competences of nursing students or professionals. Less frequently the “Organizational & Human Resources” (*n* = 2; Heffernan et al. 2022; Tzenetidis et al. 2023) in relation to structural, organizational or systemic interventions aimed at optimizing care or supporting service users within healthcare systems. Only one empty review considered “Concepts” (*n* = 1; Bertocchi et al. 2025) as relevant to the nursing care, as the relationship between standardized terminologies and missed care.

Finally, as a fifth pattern, five studies explicitly reported the absence of funding for the development of their research (Bertocchi et al. 2025; Heffernan et al. 2022; Johnson et al. 2024; Jonckers et al. 2025; Moyo et al. 2020). However, in other reviews, funding details were either unclear or not provided (*n* = 5; Brown et al. 2021; Leighton et al. 2021; Sibley et al. 2024; Tzenetidis et al. 2023; Walsh et al. 2021). Cochrane groups were an important source of funding in two studies (Chang et al. 2015; Moore et al. 2016). Academic and national institutions also played a crucial role. La Trobe University (Moyo et al. 2020), the Royal College of Surgeons in Ireland, the University of Manchester (Moore et al. 2016), and the National Institute for Health and Care Research (Heffernan et al. 2022) provided targeted funding for specific studies. In addition, independent research networks and non‐profit organizations, such as the Vermont Oxford Network (Walsh et al. 2021) and the Stichting Kinderen Kankervrij (Coyne et al. 2016), also emerged.

### 3.3 Gaps

In relation to “Clinical Practice & Patient Outcomes,” the following gaps were documented:

- Infant and maternal domain: no evidence was found on the effectiveness of specialized neonatal transport teams (Chang et al. 2015), isolation or cohort practices in neonatal units (Hanna et al. 2023), humidification of incubator air (Jonckers et al. 2025), interventions to promote shared decision making in children with cancer (Coyne et al. 2016), and the association between nurses' work environment and severe maternal morbidity (Brown et al. 2021);
- psychiatric nursing domain: no evidence was found for nurse and patient outcomes (Moyo et al. 2020), for the administration of psychotropic drugs in dementia care (Walsh et al. 2021), and for the effectiveness of community‐based interventions to improve mental health and wellbeing (Harrison et al. 2023);
- bed rest domain: no evidence was found for the effects of bed rest on healing of pressure ulcers in wheelchair users (Moore et al. 2016).

Regarding the empty reviews on “Education & Training,” Sibley et al. (2024) reported a lack of validation for Objective Structured Clinical Examinations (OSCEs) and standardized patient simulations in nurse education, while Leighton et al. (2021) found no studies that used validated instruments to assess the outcomes of traditional clinical experiences in pre‐licensure nurse education and no studies were found that addressed the preparation of nurses for transition to academic roles (Johnson et al. 2024).

In the topic “Organisational and Human Resources,” no evidence was found for the effectiveness of tri‐response models for mental health crises (Heffernan et al. 2022) or the use of validated instruments such as the Copenhagen Psychosocial Questionnaire to assess nurses' work environment and performance (Tzenetidis et al. 2023).

Regarding the “Concepts,” no empirical studies were found on the use of standardized nursing terminologies to manage unfinished nursing care (Bertocchi et al. 2025).

### 3.4 Research Recommendations

Three main recommendations were addressed in the included empty reviews, relating to (1) methodology, (2) prioritization and (3) funding (Table 3). Well‐designed primary studies, particularly for conducting randomized controlled trials and rigorous observational research, were recommended by several authors (Chang et al. 2015; Coyne et al. 2016; Hanna et al. 2023; Heffernan et al. 2022; Johnson et al. 2024; Moyo et al. 2020; Sibley et al. 2024). In addition, the development and validation of measurement tools and theoretical frameworks were identified as crucial steps to strengthen the methodological foundation of nursing research (Bertocchi et al. 2025; Leighton et al. 2021; Moore et al. 2016; Tzenetidis et al. 2023). Finally, two reviews emphasized the value of innovative and unobtrusive data collection methods, particularly in studies focusing on user perceptions and psychosocial phenomena, to evaluate community‐based interventions (Harrison et al. 2023; Heffernan et al. 2022).

**TABLE 3:** Recommendations from included empty reviews according to modified PAGER framework (Bradbury‐Jones et al. 2022).

Authors	Methodology	Priorities	Funding
Bertocchi et al. (2025)	To conduct empirical studies linking SNTs and UNC	To explore the integration of digital systems and clinical terminologies to detect, document, and prevent UNC	
Brown et al. (2021)	To conduct high‐quality cross‐sectional and longitudinal studies and use theoretical frameworks	To investigate the relation between the nurse work environment and SMM in HICs	To highlight the value of empty reviews in identifying funding priorities
Chang et al. (2015)	To conduct cluster RT among different specialist team members	To develop knowledge regarding clinical outcomes and effectiveness of specialist‐trained neonatal transport teams	
Coyne et al. (2016)	To conduct RCT to investigate the effects of interventions	To promote participation in SDM for children with cancer; to identify how new multi‐media innovations can support information exchange between children and healthcare professionals	
Hanna et al. (2023)	To conduct well‐designed randomized studies, both cluster‐randomized and individually randomized	To evaluate the effectiveness, safety, and cost‐effectiveness of patient isolation measures	
Harrison et al. (2023)	To conduct studies considering the development or adaptation of existing methods	To gather perceptions of the community‐based interventions to improve mental health and wellbeing	To generate evidence to support funding of community‐based interventions
Heffernan et al. (2022)	To conduct robust studies specifically on tri‐response models	To assess the impact of tri‐response models on reducing involuntary detentions and improving emergency mental health care	
Johnson et al. (2024)	To conduct high‐quality research studies	To explore the transition of APRNs from clinical practice to online/hybrid academia and strategies to support it	
Jonckers et al. (2025)	To conduct studies comparing humidity levels and durations in incubators	To define outcomes like thermoregulation, skin integrity, infections	
Leighton et al. (2021)	To conduct scientific research and test different models in nursing education	To develop further studies in interprofessional learning and practice for increasingly complex healthcare environments and patient care	To find available funding to support rigorous inquiry
Moore et al. (2016)	To conduct RCTs available to support or refute the use of bed rest for pressure ulcer healing in wheelchair users To ensure sufficiently large sample sizes To use standardized and validated outcome measures	To include patient‐centered outcomes To incorporate economic evaluations	
Moyo et al. (2020)	To conduct rigorous observational and experimental studies on skill mix in mental health inpatient settings	To explore appropriate outcomes such as readmission or untoward incidents	
Sibley et al. (2024)	To conduct comparative, rigorous RCTs on OSCE and SP simulations in FNP education To ensure large sample sizes	To build evidence to influence policy and practice in competency‐based education	To support funding opportunities addressing critical gap in nursing
Tzenetidis et al. (2023)	To include studies in additional languages To consider partial‐use studies	To use the entire COPSOQ questionnaire to fully assess psychosocial risks among nurses	
Walsh et al. (2021)	To conduct studies on nurses' decision‐making for psychotropic use in acute care	To evaluate effects of educational interventions on PRN administration; to investigate links between patient safety and PRN use To explore alternative non‐pharmacological strategies	

Abbreviations: APRN, Advanced Practice Registered Nurse; COPSOQ, Copenhagen Psychosocial Questionnaire; FNP, Family Nurse Practitioner; HIC, high income countries; OSCE, Objective Structured Clinical Examination; PAGER, Patterns, Advances, Gaps, Evidence for practice, and Research recommendations; PRN, pro re nata; RCT, randomized controlled trials; RT, randomized trials; SDM, shared decision‐making; SMM, severe maternal morbidity; SNT, standardized nursing terminologies; SP, standardized patients; UNC, unfinished nursing care.

In terms of priorities, empty reviews emphasized the need to expand the evidence base in relation to clinical practises for infant and maternal health (Brown et al. 2021; Chang et al. 2015; Coyne et al. 2016; Hanna et al. 2023; Jonckers et al. 2025), psychiatric care (Harrison et al. 2023; Heffernan et al. 2022; Moyo et al. 2020; Walsh et al. 2021), and interventions for prolonged bed rest (Moore et al. 2016). More research in education and training was also recommended, focusing on the validation of assessment methods such as OSCEs, simulation‐based strategies for nurse education (Sibley et al. 2024), traditional clinical placements in undergraduate nursing programs (Leighton et al. 2021), and the transition of advanced practise nurses from clinical practise to online/hybrid academia (Johnson et al. 2024). Research is needed on the structure and impact of different organizational models in psychiatry (Heffernan et al. 2022) and on the relationship between the psychosocial work environment and nurse performance using a validated instrument (Tzenetidis et al. 2023). In addition, stronger empirical foundations and validation of nursing‐specific frameworks, such as standardized terminologies (Bertocchi et al. 2025), should be studied.

In relation to funding, Sibley et al. (2024) argued that empty reviews highlight critical research gaps and can be used strategically to advocate for funding for nursing science. In line with this, Brown et al. (2021) suggested that identifying an empty review can help funding organizations to set future research priorities. Furthermore, substantial funding was found to be necessary to support rigorous investigation (Leighton et al. 2021), while Harrison et al. (2023) emphasized the need to provide funding for community‐based research interventions.

## 4 Discussion

A total of 15 systematic empty reviews were identified, all published in the last 10 years, suggesting an average publication rate of one to two reviews per year. This concentration in recent years may reflect a growing scientific interest in the importance of systematically identifying and mapping evidence gaps to support future research. At the same time, this may also point to the ongoing challenges in securing wider acceptance of empty reviews in academic publishing, as previously discussed by Gray (2021).

The geographical distribution of authors' affiliations indicates a predominance of papers from high‐income, English‐speaking countries, with most publications appearing in nursing journals and Cochrane databases. Considering that an estimated 80 systematic reviews were published per day in 2019 (Hoffmann et al. 2014) and that in PubMed the string “nursing” and “review” with a filter “Systematic reviews” yields 23,953 hits in the last 10 years (search attended on 5th February 2025), the number of empty reviews in the field of nursing appears to be very limited.

A few reviews indicated in the title that they were “empty,” which made it difficult to identify the studies in question. The authors used different terms to describe the lack of eligible studies, for example, “no eligible studies,” “*no results*,” “no studies were included” and similar. This inconsistency in terminology was noted by Lang et al. (2007) and later by other authors who argued for the introduction of standardized language (e.g., “no suitable studies found”) to improve the comprehensibility of empty reviews (Schlosser and Sigafoos 2009; Slyer 2016; Yaffe et al. 2012), enhance transparent reporting, and inform readers efficiently.

In general, authors describe the reasons for excluding studies, whether related to methods, population, or results, as well as the search strategy used. They often point out limitations in the process, such as excluding certain databases or grey literature. They also acknowledge the possibility that papers were overlooked or that a different methodology, such as a scoping review, might have been more appropriate. Overall, authors tend to clearly state both the reasons for excluding certain studies and the limitations associated with their methodology.

### 4.1 Patterns

In terms of methodology, the empty reviews were mainly conducted systematically, while a few used the scoping review method, which can identify a broader body of relevant literature, particularly in emerging or under‐researched areas (Heffernan et al. 2022; Moyo et al. 2020). The inherent flexibility of scoping reviews allows for the inclusion of different study designs, potentially reducing the occurrence of empty reviews caused by overly restrictive inclusion criteria. In addition, all retrieved reviews followed established guidelines such as the PRISMA guidelines (Page et al. 2021), the Cochrane Handbook for Systematic Reviews of Interventions (Higgings et al. 2024) and the Joanna Briggs Institute Reviewers' Manual (Aromataris et al. 2024). However, rigor in the context of empty reviews remains undefined as there are currently no formal recommendations for their reporting and quality assessment. This lack of standardized guidelines for the development and reporting of empty reviews was first noted by Montgomery et al. (Montgomery et al. 2011) and later echoed by Yaffe et al. (2012), Slyer (2016) and Gray (2021). The introduction of specific guidelines could enhance credibility by confirming the existence of a genuine absence of evidence and distinguishing truly empty reviews from those affected by methodological flaws or incomplete searches.

Almost half of the reviews we analyzed had formally registered their protocols. Preparing and registering a protocol prior to conducting the review, in which the rationale, search strategies and inclusion criteria are clearly stated, ensures transparency and minimizes potential bias (Heffernan et al. 2022; Montgomery et al. 2011). However, protocols were registered on different platforms, which introduces a degree of heterogeneity that may further complicate the identification and tracking of empty reviews. In addition, some reviews were restrictive in terms of databases, while others considered up to nine databases, which may result in the former unintentionally limiting the inclusion of studies, while the latter may be disproportionately resource‐intensive with the result that no studies are identified (FitzGerald 2018).

In terms of topics, clinical practise and patient outcomes were the most frequently addressed, while infant and maternal care and mental health interventions were most often reported as lacking, suggesting that these areas are potentially marginalized (Slyer 2016; Wells et al. 2018), which affects the ability to develop clinical guidelines (Leat 2020). The education and training of nurses have also been an issue, with studies examining both prelicensure programs and advancement in nursing. Empty reviews can serve as important indicators of research gaps, guiding future studies to promote health equity (Jones 2022) and shape research agendas (Gray 2021).

Finally, a third of the empty reviews explicitly stated that they had not received funding, just over a third acknowledged that they had, while in the remaining cases, this information was not clearly disclosed. The availability of funding can also impact subsequent publication strategies, such as publishing as open access or as reviews commissioned by academic agencies. This may influence published reviews and lead to new or less conventional areas of nursing research being neglected. However, it may also stimulate funding to support targeted studies to fill gaps in funding allocation (Moyo et al. 2020; Yaffe et al. 2012). Funding can also encourage international collaboration, which may increase the number of studies included in the results and help prevent language limitations (Gui et al. 2019).

### 4.2 Gaps

Overall, the list of identified gaps could be a priority area for researchers. Making this list visible through special registers containing only empty reviews, which are difficult to publish, could help young researchers and PhD students recognize gaps in knowledge (Gray 2021; Slyer 2016). Moreover, the absence of primary studies may reflect different conditions with distinct implications for knowledge production. First, an ontological absence occurs when, after a rigorous and comprehensive search, no relevant studies are identified because none exist. This indicates that the topic under investigation represents an emerging or neglected area within nursing science that has not yet attracted empirical attention. In such cases, the empty review serves a function by making visible what is currently unknown, helping to delineate the boundaries of the field and highlighting new areas for investigation. Second, relevant studies may exist but remain inaccessible, undiscovered, or omitted due to limitations in search strategy, database indexing, or publication practices. Here, the absence of evidence reflects limitations of the knowledge system itself rather than a true gap in empirical investigation. Third, an absence occurs when existing studies are excluded due to overly restrictive inclusion criteria, such as an exclusive focus on randomized controlled trials or high‐level quantitative designs. Although these criteria may be justified by methodological rigor, they can unintentionally narrow the evidence base and overlook relevant findings from qualitative, observational, or mixed methods research, particularly in complex areas of nursing practise.

Reviews are typically an important stage in the research process. By starting from an advanced point, such as building on an empty review that identifies a gap, researchers can accelerate evidence generation in key areas (Saiani and De Marinis 2025). Making empty reviews transparent and accessible in a dedicated registry could encourage their citation and inform future authors of primary studies when new evidence is being generated in the field. Additionally, the registry could help identify neglected areas for future research and guide equitable funding allocations. This approach aligns with broader efforts to improve research transparency and reduce bias across healthcare disciplines. As a result, the cited review would no longer be considered “empty.”

### 4.3 Research Recommendations

Consistent with the absence of studies, empty reviews highlight the need for further research and often identify focus areas that reflect existing gaps. Therefore, an empty nursing review generally concludes by stating that additional studies are needed in specific areas. After this significant effort, it is important to conduct follow‐up evaluations over time to assess whether these recommendations have been adopted by the community. In addition, such recommendations should be considered by scientific societies or international agencies working in the relevant areas as concerns that deserve prioritization.

A review always begins with a clinical or practical question; if a given question remains unanswered—methodological issues aside—it suggests a neglected area of practice that needs to be addressed to provide guidance to clinicians. Furthermore, the funding implications are also noteworthy, as empty reviews could influence future research investment and funding decisions.

### 4.4 Linking Evidence to Action

Considering these reflections, empty reviews should be understood not as endpoints but as dynamic research signals with the potential to reorient scholarly attention and resource allocation toward areas currently excluded from mainstream evidence production. To ensure these gaps do not remain invisible within academic and policy contexts, it is essential to define clear and actionable directions that could be adopted by researchers, institutional programs, funding bodies, and journal editors. To this end, and to facilitate the translation of our findings into strategic action capable of influencing future research in nursing, we summarized the key actions into a set of concise evidence recommendations (Table 4).

**TABLE 4:** Linking evidence to action.

Action item	Evidence emerged
Creating and maintaining an open registry of empty reviews with persistent identifiers	Empty reviews are difficult to identify and are underreported; a registry may make research gaps visible and guide priority identification
Standardizing terminologies and reporting of empty reviews, with explicit reasons for study exclusions	Inconsistent terminologies (“no eligible,” “no studies”) may hinder retrieval; standardized language and checklists may improve accuracy and transparency
Requiring prospective protocol registration for all reviews	Heterogeneous registration forms and post hoc changes undermine credibility and bias minimization
Expanding search strategies to include multiple databases, trial registries, grey literature, and non‐English sources, with librarian support	Restrictive sources and language limits may cause “false emptiness”; broader, librarian‐assisted searches may prevent missed studies
Using scoping reviews or mixed‐methods reviews for emergent topics instead of applying restrictive RCT‐based inclusion criteria	Overly narrow inclusion criteria lead to empty findings; scoping designs map fields and inform subsequent primary studies

Abbreviation: RCT, randomized clinical trial.

### 4.5 Limitations

This scoping review has several limitations. First, the search strategy was limited to four databases. Although these databases provide comprehensive coverage of the nursing literature, excluding additional sources may have limited the identification of relevant studies. Second, the variability of topics in the included empty reviews makes it difficult to synthesize the results. Third, the exclusion of near‐empty reviews—defined as reviews categorized as near‐empty but containing minimal or borderline evidence (Yaffe et al. 2012) may have further limited the scope of the analysis. Including near‐empty reviews could have provided additional insight into how methodological and contextual factors contribute to evidence gaps in nursing research. Finally, no formal quality assessment was conducted. We did not consider this step applicable, as there is currently no validated tool specifically for assessing the quality of empty reviews. Existing assessment tools for systematic reviews usually require an assessment of study methodologies and results, which are not available for empty reviews.

## 5 Conclusion

A total of 15 empty systematic reviews in the nursing field were identified, all published within the last 10 years, averaging one to two publications per year. Empty reviews are increasingly recognized as valuable tools for identifying areas where evidence is lacking. However, their publication is not as frequent in the nursing discipline. To better acknowledge their value, the establishment of a dedicated registry for empty reviews is recommended. Such a registry could provide information on research priorities by transparently identifying gaps in knowledge while mitigating publication bias by ensuring these reviews are accessible beyond traditional journal platforms.

From a methodological perspective, recognizing empty reviews as valuable outputs requires the development of specific guidelines for their reporting and assessment. Identifying an absolute knowledge gap is a major responsibility that must be approached with methodological rigor. Customized guidelines would not only improve the quality of empty reviews but also increase their chances of publication. In addition, pre‐registration of reviews protocols should be required to prevent retrospective changes to objectives and reduce potential bias, thereby increasing credibility and transparency.

The findings from retrieved empty reviews indicate four main priorities: clinical outcomes, education, organization, and conceptual framework. The analysis also revealed a concentration of empty reviews in certain subfields of nursing, particularly infant and maternal nursing and mental health interventions. This may indicate a systematic underrepresentation of these areas, likely influenced by prevailing publication dynamics that favor high‐impact, intervention‐focused research. Recognizing the strategic importance of empty reviews in uncovering such blind spots can support more targeted and equitable research initiatives.

In areas with limited financial support, empty reviews may identify where there is limited capacity to conduct large‐scale, high‐quality studies. Recognizing empty reviews as legitimate scientific outputs can help achieve a more balanced distribution of research funding and ensure that underfunded and under‐researched areas receive the attention and resources needed for robust evidence development. Therefore, empty reviews should not be seen as a methodological failure, but as a strategic tool to guide evidence generation and identify neglected research areas that require adequate funding.

## Funding

The PhD program of CM was funded by the European Union NextGenerationEU, National Recovery and Resilience Plan (NRRP) Mission 4 Component 1 Investment/Sub‐Investment 4.1‐Ministerial Decree No. 118 of 2 March 2023.

## Disclosure

Protocol Registration: In addition, the research protocol was registered in the Open Science Framework database (https://doi.org/10.17605/OSF.IO/KXZB4) on 8 April 2025.

## Conflicts of Interest

The authors declare no conflicts of interest.

## Supporting information


**Table S1:** Preferred Reporting Items for Systematic reviews and Meta‐Analyses extension for Scoping Reviews Checklist (Tricco et al. 2018).
**Table S2:** MeSH terms.
**Table S3:** Research strings, 5th February 2025.
**Table S4:** Characteristics of excluded studies.

## Data Availability

Data sharing not applicable to this article as no datasets were generated or analyzed during the current study.

## References

[wvn70125-bib-0001] Ahmed, S. K. , R. A. Mohammed , A. J. Nashwan , et al. 2025. “Using Thematic Analysis in Qualitative Research.” Journal of Medicine, Surgery, and Public Health 6: 100198. 10.1016/j.glmedi.2025.100198.

[wvn70125-bib-0002] Álvarez‐Bornstein, B. , and M. Montesi . 2021. “Funding Acknowledgements in Scientific Publications: A Literature Review.” Research Evaluation 29, no. 4: 469–488. 10.1093/reseval/rvaa038.

[wvn70125-bib-0003] Arksey, H. , and L. O'Malley . 2005. “Scoping Studies: Towards a Methodological Framework.” International Journal of Social Research Methodology 8, no. 1: 19–32. 10.1080/1364557032000119616.

[wvn70125-bib-0004] Aromataris, E. , C. Lockwood , K. Porritt , B. Pilla , and Z. Jordan . 2024. JBI Manual for Evidence Synthesis. JBI. 10.46658/JBIMES-24-01.

[wvn70125-bib-0005] Bercier, M. L. , and B. R. Maynard . 2015. “Interventions for Secondary Traumatic Stress With Mental Health Workers: A Systematic Review.” Research on Social Work Practice 25, no. 1: 81–89. 10.1177/1049731513517142.

[wvn70125-bib-0006] Bertocchi, L. , S. Chiappinotto , and A. Palese . 2025. “Exploring the Nexus Between the Standardized Nursing Terminologies and the Unfinished Nursing Care Phenomenon: An Empty Systematic Review.” International Journal of Nursing Knowledge 36, no. 1: 81–89. 10.1111/2047-3095.12465. 38562121

[wvn70125-bib-0007] Bradbury‐Jones, C. , H. Aveyard , O. R. Herber , L. Isham , J. Taylor , and L. O'Malley . 2022. “Scoping Reviews: The PAGER Framework for Improving the Quality of Reporting.” International Journal of Social Research Methodology 25, no. 4: 457–470. 10.1080/13645579.2021.1899596.

[wvn70125-bib-0008] Brown, K. K. , J. G. Smith , R. L. Jeffers , and C. Jean Pierre . 2021. “Association Between Nurse Work Environment and Severe Maternal Morbidity in High‐Income Countries: A Systematic Review and Call to Action.” Journal of Advanced Nursing 77, no. 3: 1206–1217. 10.1111/jan.14672. 33245160

[wvn70125-bib-0009] Chang, A. S. , A. Berry , L. J. Jones , and S. Sivasangari . 2015. “Specialist Teams for Neonatal Transport to Neonatal Intensive Care Units for Prevention of Morbidity and Mortality.” Cochrane Database of Systematic Reviews 2015, no. 10: CD007485. 10.1002/14651858.CD007485.pub2. 26508087 PMC9239562

[wvn70125-bib-0010] Cooper, H. M. 2017. Research Synthesis and Meta‐Analysis: A Step‐by‐Step Approach. 5th ed. Sage.

[wvn70125-bib-0011] Coyne, I. , D. P. O'Mathúna , F. Gibson , L. Shields , E. Leclercq , and G. Sheaf . 2016. “Interventions for Promoting Participation in Shared Decision‐Making for Children With Cancer.” Cochrane Database of Systematic Reviews 2016, no. 11: CD008970. 10.1002/14651858.CD008970.pub3. PMC6734120 27898175

[wvn70125-bib-0012] FitzGerald, D. 2018. “1710f Empty Reviews: How, Why or Why Not?” Epidemiology, A128.2‐A128. 10.1136/oemed-2018-ICOHabstracts.364.

[wvn70125-bib-0013] Florides, G. A. , and P. Christodoulides . 2023. “Factors Affecting the Independence and Reliability of Science and How These Are Perceived.” SN Social Sciences 3, no. 2: 43. 10.1007/s43545-023-00628-4. 36820304 PMC9931168

[wvn70125-bib-0014] Gray, R. 2020. “Why Do All Systematic Reviews Have Fifteen Studies?” Nurse Author & Editor 30, no. 4: 27–29. 10.1111/nae2.8.

[wvn70125-bib-0015] Gray, R. 2021. “Empty Systematic Reviews: Identifying Gaps in Knowledge or a Waste of Time and Effort?” Nurse Author & Editor 31, no. 2: 42–44. 10.1111/nae2.23.

[wvn70125-bib-0016] Gui, Q. , C. Liu , and D. Du . 2019. “Globalization of Science and International Scientific Collaboration: A Network Perspective.” Geoforum 105: 1–12. 10.1016/j.geoforum.2019.06.017.

[wvn70125-bib-0017] Hanna, M. , R. Shah , L. Marquez , R. Barzegar , A. Gordon , and M. Pammi . 2023. “Infant Isolation and Cohorting for Preventing or Reducing Transmission of Healthcare‐Associated Infections in Neonatal Units.” Cochrane Database of Systematic Reviews 2023, no. 6: CD012458. 10.1002/14651858.CD012458.pub2. PMC10297826 37368649

[wvn70125-bib-0018] Harrison, C. R. , N. Leonard , and J. Kidger . 2023. “Unobtrusive and Acceptable Ways to Gather Participant Perceptions of Community‐Based Interventions and Their Effectiveness at Improving Mental Health and Wellbeing: A Literature Review of Peer Reviewed and Grey Literature.” Health & Social Care in the Community 2023: 1–9. 10.1155/2023/1466200.

[wvn70125-bib-0019] Heffernan, J. , E. McDonald , E. Hughes , and R. Gray . 2022. “Tri‐Response Police, Ambulance, Mental Health Crisis Models in Reducing Involuntary Detentions of Mentally Ill People: A Systematic Review.” Nursing Reports 12, no. 4: 1004–1013. 10.3390/nursrep12040096. 36548169 PMC9785608

[wvn70125-bib-0020] Higgings, J. P. T. , J. Thomas , J. Chandler , et al. 2024. Cochrane Handbook for Systematic Reviews of Interventions Version 6.5 (Updated August 2024). Cochrane. www.training.cochrane.org/handbook.

[wvn70125-bib-0021] Hirt, J. , T. Nordhausen , T. Fuerst , H. Ewald , and C. Appenzeller‐Herzog . 2024. “Guidance on Terminology, Application, and Reporting of Citation Searching: The TARCiS Statement.” BMJ 385: e078384. 10.1136/bmj-2023-078384. 38724089

[wvn70125-bib-0022] Hoffmann, T. C. , P. P. Glasziou , I. Boutron , et al. 2014. “Better Reporting of Interventions: Template for Intervention Description and Replication (TIDieR) Checklist and Guide.” BMJ 348: g1687. 10.1136/bmj.g1687. 24609605

[wvn70125-bib-0023] Johnson, J. , S. Schroetter , L. Cordova , and M. Cogan . 2024. “Advanced Practice Nurses' Preparation: Transitioning From Clinical Practice to Academia: A Scoping Review.” General Submissions: Academic Settings and Education‐Based Materials. 44. https://www.sigmarepository.org/general_submissions_asem/44.

[wvn70125-bib-0024] Jonckers, T. , K. Ruhe , A. Giezen , A. Van Den Hoogen , and J. Wielenga . 2025. “Humidification of Incubator Air for Premature Infants: An Empty Systematic Review.” Journal of Neonatal Nursing 31, no. 1: 1–5. 10.1016/j.jnn.2024.07.010.

[wvn70125-bib-0025] Jones, K. 2022. Empty Review Evaluation: Update Report. Cochrane Community. https://community.cochrane.org/sites/default/files/uploads/Cochrane%20Community%20news_Empty%20review%20evaluation%20update_Feb%202022_KLJones.pdf.

[wvn70125-bib-0026] Lang, A. , N. Edwards , and A. Fleiszer . 2007. “Empty Systematic Reviews: Hidden Perils and Lessons Learned.” Journal of Clinical Epidemiology 60, no. 6: 595–597. 10.1016/j.jclinepi.2007.01.005. 17493517

[wvn70125-bib-0027] Leat, S. 2020. “2020 CAO Clinical Practice Guideline: Optometric Low Vision Rehabilitation FULL GUIDELINES.” Canadian Journal of Optometry 82, no. 1: 19–62. 10.15353/cjo.v82i1.1636.

[wvn70125-bib-0028] Leighton, K. , S. Kardong‐Edgren , A. M. McNelis , C. Foisy‐Doll , and E. Sullo . 2021. “Traditional Clinical Outcomes in Prelicensure Nursing Education: An Empty Systematic Review.” Journal of Nursing Education 60, no. 3: 136–142. 10.3928/01484834-20210222-03. 33657230

[wvn70125-bib-0029] Montgomery, P. , J. Yaffe , S. Hopewell , and L. Shepard . 2011. Running on Empty: The Cochrane Empty Reviews Project Report of Findings and Consensus Group Feedback. Empty Reviews Project Group. https://emptyreviews.wordpress.com/wp‐content/uploads/2011/11/madridmeetingpresentation‐finalpostedits.pdf.

[wvn70125-bib-0030] Moore, Z. E. , M. van Etten , and J. C. Dumville . 2016. “Bed Rest for Pressure Ulcer Healing in Wheelchair Users.” Cochrane Database of Systematic Reviews 2016, no. 10: CD011999. 10.1002/14651858.CD011999.pub2. PMC6457936 27748506

[wvn70125-bib-0031] Moyo, N. , M. Jones , D. Kushemererwa , et al. 2020. “The Association Between the Mental Health Nurse‐to‐Registered Nurse Ratio and Patient Outcomes in Psychiatric Inpatient Wards: A Systematic Review.” International Journal of Environmental Research and Public Health 17, no. 18: 6890. 10.3390/ijerph17186890. 32967198 PMC7559126

[wvn70125-bib-0032] Nicoll, L. H. , M. H. Oermann , H. Carter‐Templeton , J. K. Owens , and A. H. Edie . 2020. “A Bibliometric Analysis of Articles Identified by Editors as Representing Excellence in Nursing Publication: Replication and Extension.” Journal of Advanced Nursing 76, no. 5: 1247–1254. 10.1111/jan.14316. 32027389

[wvn70125-bib-0033] Ouzzani, M. , H. Hammady , Z. Fedorowicz , and A. Elmagarmid . 2016. “Rayyan—A Web and Mobile App for Systematic Reviews.” Systematic Reviews 5, no. 1: 210. 10.1186/s13643-016-0384-4. 27919275 PMC5139140

[wvn70125-bib-0034] Page, M. J. , J. E. McKenzie , P. M. Bossuyt , et al. 2021. “The PRISMA 2020 Statement: An Updated Guideline for Reporting Systematic Reviews.” BMJ 372: n71. 10.1136/bmj.n71. 33782057 PMC8005924

[wvn70125-bib-0035] Peters, M. D. J. , C. Marnie , H. Colquhoun , et al. 2021. “Scoping Reviews: Reinforcing and Advancing the Methodology and Application.” Systematic Reviews 10, no. 1: 263. 10.1186/s13643-021-01821-3. 34625095 PMC8499488

[wvn70125-bib-0036] Pollock, D. , M. D. J. Peters , H. Khalil , et al. 2023. “Recommendations for the Extraction, Analysis, and Presentation of Results in Scoping Reviews.” JBI Evidence Synthesis 21, no. 3: 520–532. 10.11124/JBIES-22-00123. 36081365

[wvn70125-bib-0037] Raynaud, M. , V. Goutaudier , K. Louis , et al. 2021. “Impact of the COVID‐19 Pandemic on Publication Dynamics and Non‐COVID‐19 Research Production.” BMC Medical Research Methodology 21, no. 1: 255. 10.1186/s12874-021-01404-9. 34809561 PMC8607966

[wvn70125-bib-0038] Rosati, P. , A. Crocoli , R. Saulle , et al. 2024. “Does Letting Adolescent and Young Adult Inpatients Share Decisions in Choosing the Central‐Line Insertion Site Reduce Central‐Line‐Associated Bloodstream Infections? An Empty Systematic Review.” Journal of Vascular Access 25, no. 1: 51–59. 10.1177/11297298221074448. 35114837 PMC10845812

[wvn70125-bib-0039] Saiani, L. , and M. G. De Marinis . 2025. Gli infermieri negli appelli a sostegno del Servizio Sanitario Nazionale. Assistenza Infermieristica e Ricerca. 10.1702/4514.45116. 40470853

[wvn70125-bib-0040] Schlosser, R. W. , and J. Sigafoos . 2009. “‘Empty’ Reviews and Evidence‐Based Practice.” Evidence‐Based Communication Assessment and Intervention 3, no. 1: 1–3. 10.1080/17489530902801067.

[wvn70125-bib-0041] Sheikh, A. , Y. A. Shehata , S. G. Brown , and F. E. R. Simons . 2008. “Adrenaline (Epinephrine) for the Treatment of Anaphylaxis With and Without Shock.” Cochrane Database of Systematic Reviews 2018, no. 12: CD006312. 10.1002/14651858.CD006312.pub2. PMC6517064 18843712

[wvn70125-bib-0042] Sibley, S. , K. Strout , and J. Bonnet . 2024. “Effectiveness of Objective Structured Clinical Examinations and Standardized Patient Simulations for Increasing Learner Knowledge in Family Nurse Practitioner Education: A Systematic Review of the Literature.” Nursing Education Perspectives 45, no. 6: E42–E46. 10.1097/01.NEP.0000000000001280. 38856636

[wvn70125-bib-0043] Slyer, J. T. 2016. “Unanswered Questions: Implications of an Empty Review.” JBI Database of Systematic Reviews and Implementation Reports 14, no. 6: 1–2. 10.11124/JBISRIR-2016-002934. 27532643

[wvn70125-bib-0044] Tricco, A. C. , E. Lillie , W. Zarin , et al. 2018. “PRISMA Extension for Scoping Reviews (PRISMA‐ScR): Checklist and Explanation.” Annals of Internal Medicine 169, no. 7: 467–473. 10.7326/M18-0850. 30178033

[wvn70125-bib-0045] Tzenetidis, V. , A. Kotsakis , M. Gouva , K. Tsaras , and M. Malliarou . 2023. “The Relationship Between Psychosocial Work Environment and Nurses' Performance, on Studies That Used the Validated Instrument Copenhagen Psychosocial Questionnaire (COPSOQ): An Empty Scoping Review.” Polski Merkuriusz Lekarski 51, no. 4: 417–422. 10.36740/Merkur202304117. 37756463

[wvn70125-bib-0046] Walsh, B. , S. Dahlke , H. O'Rourke , and K. F. Hunter . 2021. “Nurses' Decision‐Making Related to Administering as Needed Psychotropic Medication to Persons With Dementia: An Empty Systematic Review.” International Journal of Older People Nursing 16, no. 1: e12350. 10.1111/opn.12350. 33438810

[wvn70125-bib-0047] Wang, C. , Y. Shi , H. Lu , et al. 2022. “Global Nursing Research Activity From 2009 to 2020: A Bibliometric Analysis.” International Journal of Nursing Practice 28, no. 5: e13063. 10.1111/ijn.13063. 35599432

[wvn70125-bib-0048] Wells, S. , O. Tamir , J. Gray , D. Naidoo , M. Bekhit , and D. Goldmann . 2018. “Are Quality Improvement Collaboratives Effective? A Systematic Review.” BMJ Quality & Safety 27, no. 3: 226–240. 10.1136/bmjqs-2017-006926. 29055899

[wvn70125-bib-0049] Yaffe, J. , P. Montgomery , S. Hopewell , and L. D. Shepard . 2012. “Empty Reviews: A Description and Consideration of Cochrane Systematic Reviews With no Included Studies.” PLoS One 7, no. 5: e36626. 10.1371/journal.pone.0036626. 22574201 PMC3344923

[wvn70125-bib-0050] Železnik, D. , H. Blažun Vošner , and P. Kokol . 2017. “A Bibliometric Analysis of the *Journal of Advanced Nursing*, 1976–2015.” Journal of Advanced Nursing 73, no. 10: 2407–2419. 10.1111/jan.13296. 28295539

[wvn70125-bib-0051] Zhu, R. , M. Liu , Y. Su , X. Meng , S. Han , and Z. Duan . 2021. “A Bibliometric Analysis of Publication of Funded Studies in Nursing Research From Web of Science, 2008–2018.” Journal of Advanced Nursing 77, no. 1: 176–188. 10.1111/jan.14578. 33119957

