# Supplementary material for: Publication Dynamics Where Evidence Is Missing: Mapping Empty Reviews in Nursing
Source: Worldviews Evid Based Nurs. 2026 Feb 28;23(1):e70125. doi: 10.1111/wvn.70125 (PMC12949488; doi:10.1111/wvn.70125)
Supplement: Supplementary file 1 — Table S1: Preferred Reporting Items for Systematic reviews and Meta‐Analyses extension for Scoping Reviews Checklist (Tricco et al. 2018). Table S2: MeSH terms. Table S3: Research strings, 5th February 2025. Table S4: Characteristics of excluded studies. [file WVN-23-0-s001.docx]

## Supplementary Table 1. Preferred Reporting Items for Systematic reviews and Meta-Analyses extension for Scoping Reviews Checklist (Tricco et al., 2018)

| **SECTION** | **ITEM** | **PRISMA-ScR CHECKLIST ITEM** | **REPORTED SECTION** |
| --- | --- | --- | --- |
| **TITLE** | | | |
| Title | 1 | Identify the report as a scoping review. | Title |
| **ABSTRACT** | | | |
| Structured summary | 2 | Provide a structured summary that includes (as applicable): background, objectives, eligibility criteria, sources of evidence, charting methods, results, and conclusions that relate to the review questions and objectives. | Abstract |
| **INTRODUCTION** | | | |
| Rationale | 3 | Describe the rationale for the review in the context of what is already known. Explain why the review questions/objectives lend themselves to a scoping review approach. | Introduction |
| Objectives | 4 | Provide an explicit statement of the questions and objectives being addressed with reference to their key elements (e.g., population or participants, concepts, and context) or other relevant key elements used to conceptualize the review questions and/or objectives. | Objective of the study  Materials and Methods, Stage 1. Research Question |
| **METHODS** | | | |
| Protocol and registration | 5 | Indicate whether a review protocol exists; state if and where it can be accessed (e.g., a Web address); and if available, provide registration information, including the registration number. | Materials and Methods |
| Eligibility criteria | 6 | Specify characteristics of the sources of evidence used as eligibility criteria (e.g., years considered, language, and publication status), and provide a rationale. | Materials and Methods, Stage 2. Relevant Studies |
| Information sources | 7 | Describe all information sources in the search (e.g., databases with dates of coverage and contact with authors to identify additional sources), as well as the date the most recent search was executed. | Materials and Methods, Stage 2. Relevant Studies |
| Search | 8 | Present the full electronic search strategy for at least 1 database, including any limits used, such that it could be repeated. | Materials and Methods, Stage 2. Relevant Studies and Supplementary Table 2, 3, 4 |
| Selection of sources of evidence | 9 | State the process for selecting sources of evidence (i.e., screening and eligibility) included in the scoping review. | Materials and Methods, Stage 3. Study Selection |
| Data charting process | 10 | Describe the methods of charting data from the included sources of evidence (e.g., calibrated forms or forms that have been tested by the team before their use, and whether data charting was done independently or in duplicate) and any processes for obtaining and confirming data from investigators. | Materials and Methods, Stage 4. Data Extraction |
| Data items | 11 | List and define all variables for which data were sought and any assumptions and simplifications made. | Materials and Methods, Stage 4. Data Extraction |
| Critical appraisal of individual sources of evidence | 12 | If done, provide a rationale for conducting a critical appraisal of included sources of evidence; describe the methods used and how this information was used in any data synthesis (if appropriate). | Not appropriate |
| Synthesis of results | 13 | Describe the methods of handling and summarizing the data that were charted. | Materials and Methods, Stage 5. Data Collection, summary and report of the results, Table 1 |
| RESULTS | | | |
| Selection of sources of evidence | 14 | Give numbers of sources of evidence screened, assessed for eligibility, and included in the review, with reasons for exclusions at each stage, ideally using a flow diagram. | Results, Figure 1 |
| Characteristics of sources of evidence | 15 | For each source of evidence, present characteristics for which data were charted and provide the citations. | Results, Table 1 |
| Critical appraisal within sources of evidence | 16 | If done, present data on critical appraisal of included sources of evidence (see item 12). | Not appropriate |
| Results of individual sources of evidence | 17 | For each included source of evidence, present the relevant data that were charted that relate to the review questions and objectives. | Results, Table 2 and 3 |
| Synthesis of results | 18 | Summarize and/or present the charting results as they relate to the review questions and objectives. | Results, Table 2 and 3 |
| DISCUSSION | | | |
| Summary of evidence | 19 | Summarize the main results (including an overview of concepts, themes, and types of evidence available), link to the review questions and objectives, and consider the relevance to key groups. | Discussion, Table 4 |
| Limitations | 20 | Discuss the limitations of the scoping review process. | Discussion, Limitations |
| Conclusions | 21 | Provide a general interpretation of the results with respect to the review questions and objectives, as well as potential implications and/or next steps. | Conclusion |
| FUNDING | | | |
| Funding | 22 | Describe sources of funding for the included sources of evidence, as well as sources of funding for the scoping review. Describe the role of the funders of the scoping review. | Funding |

**Supplementary Table 2.** MeSH Terms

| **MeSH** | **PubMed definitions** |
| --- | --- |
| Nursing | The field of nursing care concerned with the promotion, maintenance, and restoration of health. |
|  | Year introduced: NR; date introduced: January 1, 1999 |
| Nurses | Professionals qualified by graduation from an accredited school of nursing and by passage of a national licensing examination to practice nursing. They provide services to patients requiring assistance in recovering or maintaining their physical or mental health. |
|  | Year introduced: 1967 (1965); date introduced: January 1, 1999 |
| Review [Publication Type] | An article or book published after examination of published material on a subject. It may be comprehensive to various degrees and the time range of material scrutinized may be broad or narrow, but the reviews most often desired are reviews of the current literature. The textual material examined may be equally broad and can encompass, in medicine specifically, clinical material as well as experimental research or case reports. State-of-the-art reviews tend to address more current matters. A review of the literature must be differentiated from historical article on the same subject, but a review of historical literature is also within the scope of this publication type. |
|  | Year introduced: 2008 (1966); date introduced: June 23, 1995 |

**Legend.** MeSH Medical Subject Headings; NR Not Reported

**Supplementary Table 3.** Research strings, 5^th^ February 2025

| **IDENTIFICATION OF STUDIES VIA DATABASES AND REGISTERS** | | |
| --- | --- | --- |
| **DATABASE** | **SEARCH STRING** | **RESULTS** |
| **PUBMED** | | |
| Population | "Nurses"[Mesh] OR nurs* | 122,6471 |
| Concept | ("Review" [Publication Type] OR review) AND (Empty[Title/Abstract] OR "no result*"[Title/Abstract] OR "no eligible"[Title/Abstract]) | 2,175 |
| Context | "Nursing"[Mesh] OR nursing | 964,551 |
| Complete string | ("Nurses"[Mesh] OR nurs*) AND (("Review" [Publication Type] OR review) AND (Empty[Title/Abstract] OR "no result*"[Title/Abstract] OR "no eligible"[Title/Abstract])) AND ("Nursing"[Mesh] OR nursing) | 61 |
| **SCOPUS** | | |
| Population | "Nurse" | 1,364,622 |
| Concept | "Review" AND ( "Empty" OR "no results" OR "no eligible" ) | 172,900 |
| Context | "nursing" | 2,631,427 |
| Complete string | "Nurse" AND "Review" AND ("Empty" OR "no results" OR "no eligible") AND "nursing" | 2,228 |
| **CINAHL EBSCO** | | |
| Population | "Nurs*" | 1,013,716 |
| Concept | "Review" AND ("Empty" OR "no result*" OR "no eligible") | 462 |
| Context | "nursing" | 812,580 |
| Complete string | "nurs*" AND ("Review" AND ("Empty" OR "no result*" OR "no eligible")) AND "nursing" | 50 |
| **COCHRANE** | | |
| Population | Nurs* OR [nurses] | 3,359 |
| Concept | ([review] OR Review) AND (Empty OR no NEXT result* OR no NEXT eligible) | 1,300 |
| Context | Nursing OR [nursing] | 2,453 |
| Complete string | (Nurs* OR [nurses]) AND (([review] OR Review) AND (Empty OR no NEXT result* OR no NEXT eligible)) AND (Nursing OR [nursing]) | 421 |
| **IDENTIFICATION OF STUDIES VIA OTHER METHODS** | | |
| **SOURCES** | **SEARCH STRING** | **RESULTS** |
| Citation searching | - | 0 |
| Data Station Life Sciences | "empty review" nursing | 106 |
| Google scholar | "empty review" AND nursing | 342 |
| MedRxiv | "empty review" | 4 |
| Open Science Framework | “empty review” | 29 |
| ProQuest Dissertation and Theses global | "empty review" | 6 |
| PROSPERO | “empty review” | 15 |
| Sigmarepository | "empty review" | 35 |

**Legend.** CINAHL, Cumulative Index to Nursing and Allied Health Literature; PROSPERO, International prospective register of systematic reviews

**Supplementary Table 4**. Characteristics of excluded studies

| **Study** | **Reasons for exclusion** |
| --- | --- |
| Bercier & Maynard (2015) | Focus on mental health workers, a category which, according to the authors’ definition, primarily includes social workers, psychologists, therapists, and counsellors, and not on nurses. |
| Rosati et al. (2024) | It is conceptually and methodologically framed within a multidisciplinary medical perspective, with no explicit focus on nursing theories, nursing-sensitive outcomes, or implications for nursing-specific practice or education. The review adopts a multidisciplinary clinical perspective without theoretical grounding or implications specific to nursing science or practice, which was a required eligibility criterion. |
| Sheikh et al. (2008) | The study was designed and conducted within a pharmacological and emergency medicine framework, with no explicit focus on nursing practice, nursing-sensitive outcomes, or theoretical underpinnings relevant to nursing science. It evaluates the use of adrenaline for anaphylaxis but does not explore this issue from a nursing-specific perspective nor does it contribute to knowledge development in nursing care, decision-making, or patient education. |
